# Characteristics and Outcomes of Cocaine-Related Spontaneous Intracerebral Hemorrhages

**DOI:** 10.1155/2013/124390

**Published:** 2013-03-05

**Authors:** Abubakr A. Bajwa, Scott Silliman, James D. Cury, Vandana Seeram, Adil Shujaat, Faisal Usman, Vinny Samuel

**Affiliations:** ^1^Division of Pulmonary, Critical Care and Sleep Medicine, University of Florida College of Medicine-Jacksonville and Shands Hospital, 655 West 8th Street, Jacksonville, FL 32209, USA; ^2^Department of Neurology, University of Florida College of Medicine-Jacksonville, Jacksonville, FL 32209, USA

## Abstract

To date there is only one single-center study that has exclusively reported characteristics, location, and outcomes of spontaneous intracerebral hemorrhages (ICH) among cocaine users. We aimed to describe the radiological location and characteristics along with clinical outcomes of spontaneous ICH in a similar population. We conducted a retrospective chart review of consecutive patients admitted to a tertiary care hospital, with a spontaneous ICH, who had a urine drug screen performed within 48 hours of admission. Exposure to cocaine was defined by a positive urine drug screen within 48 hours of hospital admission. Demographics, radiographic features of ICH, and short-term clinical outcomes of patients with a positive urine drug screen were analyzed and compared with the cocaine negative group. Among the 102 patients analyzed, 20 (19.6%) had documented exposure to cocaine. There was a predominance of males in both groups with significantly more Blacks in the cocaine positive group (*P* = 0.0246). A statistically significant number of patients with cocaine use had ICH in a subcortical location (*P* = 0.0224) when compared to cocaine negative patients. There was no difference in GCS, ICH volume, intraventricular extension, ICU days, hospital days, hospital cost, mortality, and ICH score. ICH in cocaine use is more frequently seen in the subcortical location.

## 1. Introduction

Cocaine use leads to multiple neurovascular complications [1]. Although stroke has been a well-recognized complication of cocaine use, there have been relatively few studies evaluating the radiological and clinical characteristics of spontaneous intracerebral hemorrhages (ICH) among cocaine users [2–12]. Until recently, most of the published studies have been autopsy series and suggested vasculitis and aneurysmal rupture as common causes of ICH [13–19]. Among various types of intracranial hemorrhages in cocaine users, perhaps the most extensively described entity has been subarachnoid hemorrhage. Giraldo et al. while evaluating ischemic and hemorrhagic strokes in African American population with crack-cocaine use showed that although ICH was more common among crack-cocaine users, they had higher odds of better functional outcomes [11]. Recently Martin-Schild et al. described a cohort of patients with ICH and found that cocaine users had higher admission blood pressure, more subcortical hemorrhages, higher mortality, and worse functional outcomes compared to nonusers [20]. Most of the studies have associated spikes in blood pressure (BP) as an important etiological factor in the development of ICH. Since the association with subarachnoid has been well described and to date there is only single center study which exclusively describes the relationship between cocaine use and ICH, we decided to analyze and compare cocaine-associated spontaneous ICH to cocaine-negative ICH population.

## 2. Methods

After the institutional review board approval, we conducted a retrospective chart review of consecutive patients admitted to the intensive care unit at Shands Jacksonville between July 2007 and July 2009 with a diagnosis of spontaneous ICH who had a urine drug screen done within 48 hours of admission. Shands Jacksonville is a 750-bed tertiary care center which has been a Joint Commission certified primary stroke center since 2006. Patients with subarachnoid hemorrhages, subdural hematomas, and ischemic stroke-related hemorrhages were excluded from analysis. Exposure to cocaine was defined as a positive urine drug screen when performed within 48 hours of admission. Patients with negative urine drug screen were used as comparison. We also collected information on other concomitant drugs detected on the urine drug screen. Data on baseline demographics, first systolic and diastolic BP documented upon arrival in ED, comorbidities (history of diabetes hypertension and coronary artery disease from medical records), and anticoagulant use were collected. Parameters collected related to ICH included ICH score calculated by evaluation of ICH volume in cm^3^, Glasgow Coma Scale (GCS), intraventricular extension, and infratentorial origin. ICH volume was calculated using ABC/2 formula after CT scans were retrospectively reviewed by the investigators [21]. The location of ICH was divided into lobar and subcortical areas. Subcortical areas included basal ganglia, thalamus, cerebellum, and brainstem including pons.

Outcomes data included ICU and hospital length of stay (LOS), cost of hospitalization, ventriculostomy placement, need for mechanical ventilation, tracheostomy, code status, and compassionate wean decision, that is, not escalating medical care, stopping life sustaining support, and allowing natural death. If a brain computed tomography angiogram (CTA) was performed, we reviewed the report to collect information regarding the presence or absence of an arterial vascular malformation (AVM).

Mean and range or standard deviation was used to describe normally distributed continuous data. Median and interquartile range (IQR 25–75) was used to describe nonnormally distributed data, and percentages were used to describe categorical data. A Fisher's exact test and chi-square test were performed to analyze the relationship between categorical variables when appropriate. A *P* value less than 0.05 was considered statistically significant. Statistical analysis was performed using JMP (Statistical Discovery by SAS, Cary, NC, USA).

## 3. Results

We screened a total of 502 patients. Patients with subarachnoid hemorrhages, subdural hematomas, ischemic stroke related hemorrhages, and ICH without a urine drug screen were excluded. A total of 102 consecutive patients met the inclusion criteria of a spontaneous ICH and a urine drug screen performed within 48 hours of admission. A total of 20 (19.6%) patients tested positive for cocaine. No patients were excluded due to a urine drug screen being performed beyond the 48 hours cutoff. In the cocaine-positive group we detected 3 patients with concomitant marijuana use, while 1 patient tested positive for only marijuana in the cocaine-negative group. Cocaine positive patients were more likely to be Black (*P* = 0.0246) and younger (*P* = 0.049); however, there was no difference in gender between the two groups. Fourteen (70%) patients in the cocaine-positive group were older than 50 with the oldest being 68 years old. There was a trend towards higher median diastolic BP among cocaine users (*P* = 0.0637) without any significant difference between systolic BP. None of the cocaine users and 4 (5%) patients in the cocaine negative group were on anticoagulation (Table 1).

No difference was identified in ICH score or various variables used to determine ICH score between the two groups (Table 2). Cocaine users were more likely to have an ICH in the subcortical location (*P* = 0.0224) when compared to the cocaine-negative group who had a higher number of lobar ICH. In both groups, the most common location in the subcortical area was basal ganglia followed by the thalamus. A brain computed tomographic angiography (CTA) was performed in 7 (41%) of cocaine-positive group and 17 (20%) of cocaine-negative group. On CTA there was only 1 patient in the cocaine-negative group who had a possible vascular malformation, which was later ruled out based on a cerebral angiogram (Table 2). 

We did not notice any significant difference among clinical outcomes which included ICU or hospital LOS, ventriculostomy placement, mechanical ventilation, need for tracheostomy, or cost of hospitalization. The decision to allow natural death or initiate comfort measures only was also not statistically different among the two groups (Table 3).

## 4. Discussion

**Figure 1:**
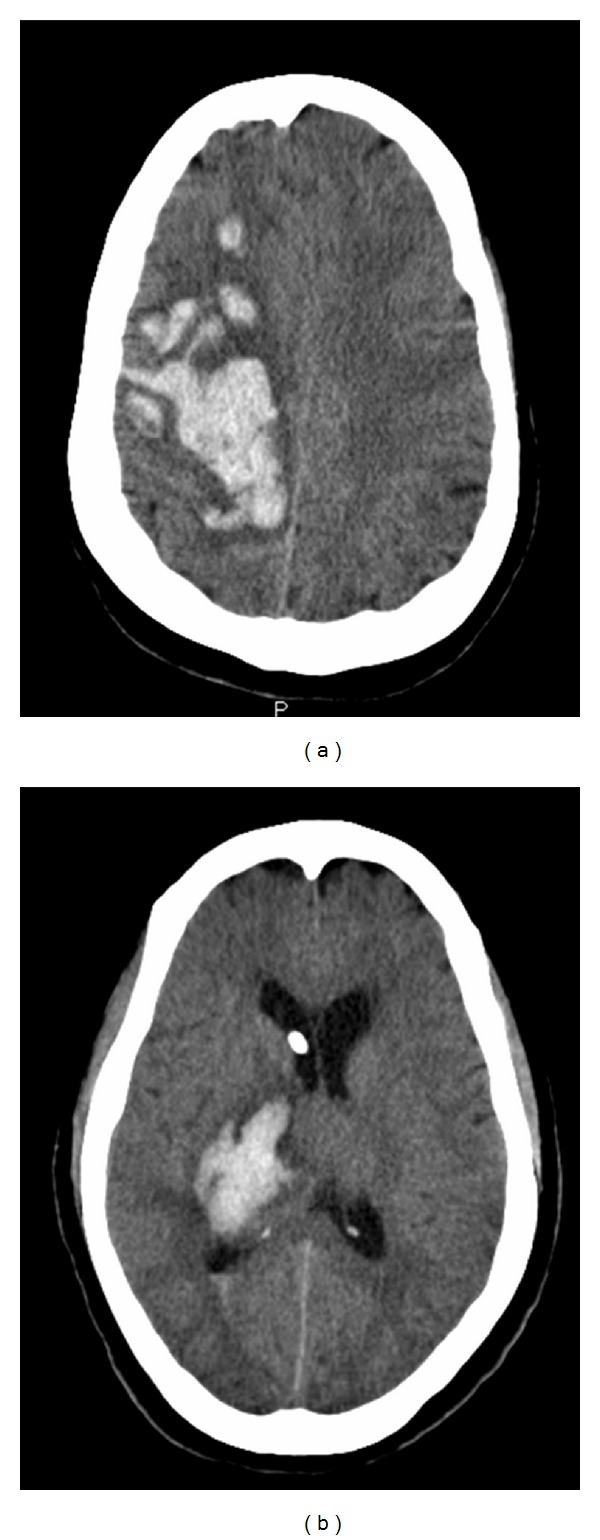
(a) Lobar (R frontoparietal lobe) ICH in a noncocaine user and (b) subcortical (R thalamus) ICH in a cocaine user.

This is one of the largest studies analyzing spontaneous ICH in active cocaine users. Our study shows that cocaine use may be fairly common among spontaneous ICH patients. There is a predilection for ICH in a subcortical location among active cocaine users. An example of a lobar ICH in a noncocaine user and subcortical ICH in a cocaine user is shown in Figure 1. Although only a small number of patients underwent CTA brain, we did not find any cases of cerebral vasculitis or AVM in the cocaine-positive group. We also did not notice any difference in hospital outcomes among cocaine-positive when compared to cocaine-negative population. Although some of the results from our study were in agreement with the recently published data in terms of ethnic distribution and higher number of ICH being in the subcortical location among active cocaine users, we did not notice a significant difference among other clinical parameters such as mortality, BP, ICH score, and LOS.

Crack-cocaine use has been linked with a variety of acute medical conditions ranging from acute coronary artery vasospasm, rhabdomyolysis, to asthma exacerbation and acute lung injury (crack lung). The neurovascular complications related to cocaine use have been gaining an increasing attention. In addition to evidence of accelerated atherosclerosis in cocaine users, it has been shown that remote use of cocaine generally leads to ischemic strokes and TIAs, while acute use mostly results in ICH [1, 22–24]. Among our cohort, cocaine-use-related ICH was seen mostly among males and across all age groups with the oldest patient in our cohort who tested positive for cocaine being 68 years old. Given the range of acute medical conditions that may impact hospital course and a broad age range among users, it may be necessary to screen for cocaine in all patients with spontaneous ICH. 

The difference in ICH location, as also seen in the study by Martin-Schild et al., is an interesting finding which does not have a very clear explanation. Our study also shows that although spontaneous ICH in a subcortical area is more common among crack-cocaine users, distribution of ICH in various subcortical structures is similar among cocaine-positive and cocaine-negative groups, with the most common area being basal ganglia followed by thalamus, brainstem, and cerebellum. Even though the overall ICH score was not that high in our study, a major component of the mortality percentage was driven by smaller volume ICH primarily in the brainstem location. The mortality in ICH varies with various topographic locations which was shown in a study done by Arboix et al. which showed that the highest mortality (65%) was seen in multiple-topographic involvement while brainstem ICH had a mortality of 40% [25]. Given that the majority of the patients in both groups had a diagnosis of systemic hypertension, a possible mechanism may be that cocaine use causes spikes in BP in patients with preexisting hypertensive cerebral vasculopathy in the subcortical areas, leading to ICH primarily in this location. A study evaluating for chronic hypertensive changes in brain in an autopsy series of 26 patients with cocaine-related ICH, 7 of 19 cases did not have any findings suggestive of chronic vasculopathy. This argues against the background of chronic hypertensive vasculopathy being the sole mechanism responsible for ICH in this population [14]. Perhaps a more reasonable hypothesis would be similar to the one proposed by Kibayashi et al. stating that a combination of cocaine-related spikes in BP, lowering of the upper limit of BP for cerebral autoregulation among cocaine users, and preexisting hypertensive cerebral vasculopathy leads to spontaneous ICH [14]. 

There are a number of limitations in our study. The retrospective design limits adequate detailed analysis. There may also have been a selection bias since we only evaluated patients who underwent urine drug screen which was approximately 20% of all ICH population evaluated at our institute. Since not all ICH patients underwent urine drug screen, prevalence of cocaine use among this population cannot be determined. Given a small number of patients in the cocaine-positive group, a number of variables could not be adequately compared. Although no AVMs were diagnosed, the number of patients who underwent CTA was very small.

Even though the study has limitations, it has important implications. Given various other coexisting acute complications that can occur as a result of cocaine use, it is perhaps reasonable to screen all patients presenting with spontaneous ICH especially the ones in the subcortical location. Further studies are needed to evaluate the exact mechanism by which cocaine use predisposes individuals to develop a spontaneous ICH.

## Figures and Tables

**Table 1:** Demographics of cocaine-positive and cocaine-negative ICH patients.

	Cocaine positive (*N* = 20)	Cocaine negative (*N* = 82)	*P* value
Age median (IQR 25–75)	51 (48–57)	57 (49–65)	0.049
Gender (male), %	80	67	0.255
Race *n*, (%)			
Black	19 (95)	53 (64.6)	0.024
White	1 (5)	25 (30.4)	0.035
Hispanic	0 (0)	2 (2.4)	0.984
Asian	0 (0)	2 (2.4)	0.984
Admission SBP, median (IQR 25–75)	209 (189–224)	199.5 (168–226)	0.098
Admission DBP, median (IQR 25–75)	120 (104–140.5)	106.5 (89–133)	0.063
History of hypertension *n*, (%)	14 (70)	68 (83)	0.526
History of diabetes *n*, (%)	1 (5)	24 (29)	0.064
History of CAD *n*, (%)	1 (5)	9 (11)	0.6844
Anticoagulation use *n*, (%)	0 (0)	4 (5)	1.000
Concomitant marijuana use *n*, (%)	3 (18)	1 (1)	0.423

CAD: coronary artery disease.

**Table 2:** ICH characteristics in cocaine-positive and cocaine-negative patients.

	Cocaine positive (*N* = 20)	Cocaine negative (*N* = 82)	*P* value
ICH score, median (min–max) (IQR)	1.5 (0–4) (1–3)	1 (0–4) 1 (1–3)	0.425
GCS, median (min–max) (IQR)	11 (3–15) (3–15)	10.5 (3–15) (6–14.5)	0.427
ICH volume, median mL^3^, median (min–max) (IQR)	6.0 (0.35–75) (3–23.34)	9.98 (0.25–180) (2.92–24.75)	0.805
Intraventricular extension, *n*, (%)	10 (50)	46 (56)	0.794
Infratentorial origin, *n*, (%)	4 (20)	9 (11)	0.227
Location, *n* (%)			
Lobar	1 (5.88)	24 (28.92)	0.0224
Subcortical	19 (95)	58 (71)	0.0294
Basal ganglia	10 (50)	36 (43.37)	
Thalamus	5 (26.3)	13 (15.66)	
Brainstem	3 (15)	6 (7.23)	
Cerebellum	1 (5)	3 (3.61)	
Computed tomography angiography performed, *n* (%)	7 (41)	17 (20)	0.238

IQR: 25%–75%, GCS: Glasgow Comas Scale, ICH: intracranial hemorrhage, IVH: intraventricular hemorrhage.

**Table 3:** Short-term outcomes of cocaine-positive and cocaine-negative patients.

	Cocaine positive (*N* = 20)	Cocaine negative (*N* = 82)	*P* value
ICU LOS, median (IQR)	3 (2–5)	3 (2–9)	0.788
Hospital LOS, median (IQR)	5.5 (3–21)	7 (3–15)	0.175
Ventriculostomy, *n* (%)	3 (15)	25 (30)	0.263
Mechanical ventilation, *n* (%)	11 (55)	61 (74)	0.279
Tracheostomy performed, *n* (%)	3 (15)	17 (21)	0.756
Mortality *n*, (%)	10 (50%)	35 (43%)	0.619
Allow natural death, *n* (%)	9 (53)	38 (46)	0.803
Compassionate wean, *n* (%)	6 (35)	24 (29)	0.588
Cost, median (IQR)	$38233 (32696–128917)	$56815 (32417–123737)	0.385

ICU: intensive care unit, LOS: length of stay, IQR: 25%–75%.

## References

[1] Toossi S, Hess CP, Hills NK, Josephson SA (2010). Neurovascular complications of cocaine use at a tertiary stroke center. *Journal of Stroke and Cerebrovascular Diseases*.

[2] Fessler RD, Esshaki CM, Stankewitz RC, Johnson RR, Diaz FG (1997). The neurovascular complications of cocaine. *Surgical Neurology*.

[3] Riggs JE, Gutmann L (1997). Crack cocaine use and stroke in young patients. *Neurology*.

[4] O'Brien CP (1998). Stroke in young women who use cocaine or amphetamines. *Epidemiology*.

[5] Petitti DB, Sidney S, Quesenberry C, Bernstein A (1998). Stroke and cocaine or amphetamine use. *Epidemiology*.

[6] Sen S, Silliman SL, Braitman LE (1999). Vascular risk factors in cocaine users with stroke. *Journal of Stroke and Cerebrovascular Diseases*.

[7] Qureshi AI, Fareed M, Suri K, Guterman LR, Hopkins LN (2001). Cocaine use and the likelihood of nonfatal myocardial infarction and stroke: data from the third national health and nutrition examination survey. *Circulation*.

[8] Treadwell SD, Robinson TG (2007). Cocaine use and stroke. *Postgraduate Medical Journal*.

[9] Westover AN, McBride S, Haley RW (2007). Stroke in young adults who abuse amphetamines or cocaine: a population-based study of hospitalized patients. *Archives of General Psychiatry*.

[10] Koch S, Sacco RL (2008). Cocaine-associated stroke: some new insights?. *Nature Clinical Practice Neurology*.

[11] Giraldo EA, Taqi MA, Vaidean GD (2010). A case-control study of stroke risk factors and outcomes in African American stroke patients with and without crack-cocaine abuse. *Neurocritical Care*.

[12] Wojak JC, Flamm ES (1987). Intracranial hemorrhage and cocaine use. *Stroke*.

[13] Daras M, Tuchman AJ, Koppel BS, Samkoff LM, Weitzner I, Marc J (1994). Neurovascular complications of cocaine. *Acta Neurologica Scandinavica*.

[14] Kibayashi K, Mastri AR, Hirsch CS (1995). Cocaine induced intracerebral hemorrhage: analysis of predisposing factors and mechanisms causing hemorrhagic strokes. *Human Pathology*.

[15] McEvoy AW, Kitchen ND, Thomas DGT (2000). Intracerebral haemorrhage in young adults: the emerging importance of drug misuse. *British Medical Journal*.

[16] Merkel PA, Koroshetz WJ, Irizarry MC, Cudkowicz ME (1995). Cocaine-associated cerebral vasculitis. *Seminars in Arthritis and Rheumatism*.

[17] Morrow PL, McQuillen JB (1993). Cerebral vasculitis associated with cocaine abuse. *Journal of Forensic Sciences*.

[18] Aggarwal SK, Williams V, Levine SR, Cassin BJ, Garcia JH (1996). Cocaine-associated intracranial hemorrhage: absence of vasculitis in 14 cases. *Neurology*.

[19] Nolte KB, Brass LM, Fletterick CF (1996). Intracranial hemorrhage associated with cocaine abuse: a prospective autopsy study. *Neurology*.

[20] Martin-Schild S, Albright KC, Hallevi H (2010). Intracerebral hemorrhage in cocaine users. *Stroke*.

[21] Kothari RU, Brott T, Broderick JP (1996). The ABCs of measuring intracerebral hemorrhage volumes. *Stroke*.

[22] Benzaquen BS, Cohen V, Eisenberg MJ (2001). Effects of cocaine on the coronary arteries. *American Heart Journal*.

[23] Kolodgie FD, Virmani R, Cornhill JF, Herderick EE, Smialek J (1991). Increase in atherosclerosis and adventitial mast cells in cocaine abusers: an alternative mechanism of cocaine-associated coronary vasospasm and thrombosis. *Journal of the American College of Cardiology*.

[24] Kolodgie FD, Wilson PS, Cornhill JF, Herderick EE, Mergner WJ, Virmani R (1993). Increased prevalence of aortic fatty streaks in cholesterol-fed rabbits administered intravenous cocaine: the role of vascular endothelium. *Toxicologic Pathology*.

[25] Arboix A, Comes E, García-Eroles L (2002). Site of bleeding and early outcome in primary intracerebral hemorrhage. *Acta Neurologica Scandinavica*.

